# Relationship between Cognitive Performance and Motor Dysfunction in Patients with Parkinson's Disease: A Pilot Cross-Sectional Study

**DOI:** 10.1155/2015/365959

**Published:** 2015-03-31

**Authors:** Valentina Varalta, Alessandro Picelli, Cristina Fonte, Stefania Amato, Camilla Melotti, Vanja Zatezalo, Leopold Saltuari, Nicola Smania

**Affiliations:** ^1^Neuromotor and Cognitive Rehabilitation Research Center, Department of Neurological and Movement Sciences, University of Verona, 37134 Verona, Italy; ^2^Department of Neurology, Hochzirl Hospital, 6170 Zirl, Austria; ^3^Research Unit for Neurorehabilitation South Tyrol, 39100 Bolzano, Italy; ^4^Neurorehabilitation Unit, Hospital Trust of Verona, 37126 Verona, Italy

## Abstract

The aim of this pilot cross-sectional study was to extensively investigate the relationships between cognitive performance and motor dysfunction involving balance and gait ability in patients with Parkinson's disease. Twenty subjects with Parkinson's disease underwent a cognitive (outcomes: Frontal Assessment Battery-Italian version, Montreal overall Cognitive Assessment, Trail Making Test, Semantic Verbal Fluency Test, and Memory with Interference Test) and motor (outcomes: Berg Balance Scale, 10-Meter Walking Test, 6-Minute Walking Test, Timed Up and Go Test performed also under dual task condition, and Unified Parkinson's Disease Rating Scale) assessment. Our correlation analyses showed that balance skills are significantly correlated with executive functions, cognitive impairment, and ability to switch attention between two tasks. Furthermore, functional mobility showed a significant correlation with cognitive impairment, verbal fluency, and ability to switch attention between two tasks. In addition, the functional mobility evaluated under the dual task condition showed a significant correlation with cognitive impairment and ability to switch attention between two tasks. These findings might help early identification of cognitive deficits or motor dysfunctions in patients with Parkinson's disease who may benefit from rehabilitative strategies. Future prospective larger-scale studies are needed to strengthen our results.

## 1. Introduction

Parkinson's disease (PD) is an idiopathic neurodegenerative disorder caused by a progressive loss of dopaminergic neurons in the substantia nigra pars compacta [1]. Clinical manifestations include reduced amplitude of movement, hypokinesia, rigidity, resting tremor, and postural instability [1, 2]. The resulting abnormal gait patterns raise the risk of falls, with up to 63% of people with PD reporting more than one fall per year [3]. In addition to motor symptoms, about 25% of newly diagnosed PD sufferers present with cognitive deficits [4] frequently involving attention, memory, visuospatial, and executive functions in nondemented people with PD [4–7]. Often, PD-associated cognitive deficits are underestimated in daily clinical practice probably because cognitive assessment is mainly based on the Mini Mental State Examination (MMSE) that has low sensitivity for detecting alterations in specific cognitive and executive functions [8–11].

Internal generation of movement and executive functions both require decision-making processes in order to select an action among several alternative possibilities for the task at hand. The basal ganglia (mostly modulated by dopaminergic projections) seem to have an important role in the mediation of cognitive and motor modules to generate an appropriate decision on a resulting action for the task being performed [12]. Patients with PD showed a significant reduction of the dopaminergic projections to the striatum [13]. Previous studies indicate that executive deficits in PD patients without dementia are associated with dysfunction of the caudate nucleus [14–16], suggesting that dopamine is involved in the transfer of information first processed in cognitive brain networks, toward motor-related networks, sequentially [12]. Other potential factors that may influence the relationship between cognitive and motor function in people with PD are depression and age [17, 18].

Although cognitive-motor relationships have been widely described in patients with PD, evaluation takes into consideration a narrow range of outcome measures which do not allow for in-depth complete assessment of cognitive deficits and motor dysfunctions in PD [3, 19–23]. Hence, a closer examination of cognitive-motor relationships is needed to better define to what extent motor aspects depend on cognitive ones and to inform the design of new treatment protocols. Therefore, the aim of this study was to extensively investigate the relationships between cognitive performance and motor dysfunction involving balance and gait ability in patients with PD.

## 2. Materials and Methods

This was a pilot cross-sectional study. Inclusion criteria were confirmed diagnosis of idiopathic PD according to the UK Brain Bank Criteria [24] and MMSE score > 24 [25]. Exclusion criteria were severe dyskinesias or on-off fluctuations, severe comprehension deficit, psychotic disorders, history of alcohol or drug abuse, deficits of somatic sensation involving the lower limbs as assessed by neurological examination, vestibular disorders or paroxysmal vertigo, and other neurological or orthopedic conditions involving the lower limbs such as musculoskeletal diseases, severe osteoarthritis, peripheral neuropathy, and joint replacement. All subjects were outpatients and gave their informed written consent to participate. The study was carried out according to the Declaration of Helsinki and approved by the Local Ethics Committee.

### 2.1. Evaluation Procedures

During the study period, subjects were instructed to take their PD medications regularly. Each subject underwent the following cognitive and motor assessments during the “on” phase (1 to 2.5 hours after having taken their morning dose). The same raters evaluated all subjects (CF and SA performed the cognitive assessment; AP and VZ performed the motor assessment) in an outpatients clinical setting.

#### 2.1.1. Cognitive Assessment

The main cognitive outcomes were the Frontal Assessment Battery-Italian version (FAB-it) [26] and the Montreal overall Cognitive Assessment (MoCA) [11].

The FAB-it assesses executive functions such as conceptualization, mental flexibility, programming, sensitivity interference, inhibitory control, and environmental autonomy. It consists of 6 tests (similarities, lexical fluency, motor series “Luria” test, conflicting instructions, go/no-go task, and prehension behavior) each rated on a scale from 0 to 3 points. The total score is the sum of all items; the range is from 0 (worst performance) to 18 (best performance) [26].

The MoCA investigates patient's skills in 5 domains: visuospatial/executive, naming, memory, attention, abstraction, and orientation. The total score is the sum of all items, with a maximum score of 30 (best performance) [11].

Other cognitive outcomes were the Trail Making Test (TMT), the Semantic Verbal Fluency Test (SVF), and the Memory with Interference (MI) Test.

Attention capacity was evaluated with the TMT (parts A and B) to assess selective attention, psychomotor speed, and sequencing skills. Part B also investigates the ability to switch attention between two rules or tasks. The time taken to complete the trails is recorded (longer = worse performance) [27].

The SVF assesses verbal fluency by determining the number of words pertaining to a specific semantic category that subjects can spontaneously generate in 2 minutes (higher = better performance) [28].

Working memory was assessed with the MI Test. Subjects are asked to recall a consonant trigram after an interval delay during which they have to count forward starting from a 3-digit number randomly presented by the examiner immediately after the trigram. At the end of an interval delay of 10 seconds, subjects have to recall the trigram. The maximum score is 9 (best performance) [29].

Besides cognitive skills, the patients' mood was evaluated through the Beck Depression Inventory. This tool consists of 21 items rated on a four-point scale of severity focusing on psychological aspects of depression. The total score is the sum of all items; the maximum score is 63 (worst mood) [30].

#### 2.1.2. Motor Assessment

The main motor outcome was performance on the Berg Balance Scale (BBS). This 14-item scale (0–4 points/task; best score = 56) evaluates balance abilities during sitting, standing, and positional changes [31].

Other motor outcomes were the 10-Meter Walking Test (10MWT), the 6-Minute Walking Test (6MWT), the Timed Up and Go Test (TUG), and the Unified Parkinson's Disease Rating Scale (UPDRS).

The 10MWT was selected as a measure of gait speed [32, 33]. The subjects were asked to walk on a flat hard floor at their fastest speed for 10 m without assistance or the use of walking aids (a 10 m walkway was marked by two lines on the floor at 2 m and at 8 m). In order to minimize acceleration and deceleration, gait speed was measured in the 6 m between the two marks (timing started when the toes of the leading foot crossed the 2 m mark and stopped when the toes of the leading foot crossed the 8 m mark) [32, 33]. Time was measured using a handheld stopwatch.

Walking capacity was assessed using the 6MWT [34]. The subjects were asked to cover as much ground as possible in 6 min (walking continuously at their possibly fastest speed without the use of walking aids) along a marked distance (1 lap, 40 m). The distance covered was recorded.

The TUG is functional mobility test associated with balance problems and falls in older adults in which a subject must stand up, walk 3 meters, turn around, walk back, and sit down. The time taken to complete the test is correlated with the level of functional mobility [35]. The subjects performed the TUG under a dual task condition in which they were also asked to count backwards from a randomly selected number between 20 and 100 (TUG-COG) [36].

The UPDRS is a validated tool to follow the longitudinal course of PD. It has 4 subsections. Part III (motor examination) was used (score ranges from 0 to 108; high = worst performance) [37].

### 2.2. Statistical Analysis

Statistical analysis was carried out using the Statistical Package for Social Sciences software, version 20.0, for Macintosh (SPSS Inc, Chicago, IL). Spearman's rank correlation analysis was performed to determine the correlation between motor and cognitive outcomes. Forward stepwise multiple linear regression analyses were performed to further clarify the relationship between cognitive outcomes (the FAB-it, the MoCA, the SVF, and the TMT-B were defined as independent variables), potential confounders (age and the BDI were defined as independent variables), and motor outcomes (the BBS, the TUG, and the TUG-COG were defined as dependent variables). The alpha level for significance was set at *P* < 0.05.

## 3. Results

Twenty subjects (12 males, 8 females; mean age 70.3 ± 6.34 years; mean years of schooling 10.65 ± 5.19) presenting with idiopathic PD (mean disease duration 9.88 ± 5.79 years) were recruited from among 46 outpatients consecutively admitted to the Neurological Rehabilitation Unit of the Azienda Ospedaliera Universitaria of Verona, Italy. The enrollment period was from January to June 2014. Raw data of patients' performance in all outcome measures are detailed in Table 1.

As to the motor-cognitive correlation in PD, the spearman's analysis showed that the BBS was significantly directly related to the FAB-it (*P* < 0.001 and *ρ* = 0.790) and the MoCA (*P* = 0.015 and *ρ* = 0.534) and it was significantly inversely related to the TMT-B (*P* = 0.005 and *ρ* = −0.597). The TUG was significantly inversely related to the MoCA (*P* = 0.045 and *ρ* = −0.564) and the SVF (*P* < 0.006 and *ρ* = −0.713) and it was significantly directly related to the TMT-B (*P* = 0.021 and *ρ* = −0.630). The TUG-COG was significantly inversely related to the MoCA (*P* = 0.025 and *ρ* = −0.667) and significantly directly related to the TMT-B (*P* < 0.020 and *ρ* = 0.683) (see Table 2).

As reported in Table 3, the multiple linear regression analysis showed a significant direct association between the BBS and the FAB-it (*P* = 0.042; *β* = 0.640).

## 4. Discussion

The aim of this pilot cross-sectional study was to perform an in-depth investigation of the relationship between cognitive deficits and motor dysfunctions involving balance and gait ability in patients with PD. We found that balance skills (as measured by the BBS) are significantly correlated with executive functions (as measured by the FAB-it), cognitive impairment (as measured by the MoCA), and ability to switch attention between two tasks (as measured by the TMT-B). Furthermore, functional mobility (as measured by the TUG) showed a significant correlation with cognitive impairment (as measured by the MoCA), verbal fluency (as measured by the SVF), and ability to switch attention between two tasks (as measured by the TMT-B). In addition, the functional mobility evaluated under the dual task condition (as measured by the TUG-COG) showed a significant correlation with cognitive impairment (as measured by the MoCA) and ability to switch attention between two tasks (as measured by the TMT-B).

Despite the fact that cognitive-motor relationships have been previously reported in PD [3, 19–23], many of these studies did not extensively investigate motor or cognitive functions [3, 19, 22, 23]. For example, Lee and colleagues examined only the postural instability skill. Specifically they investigated the relationship between postural instability, as measured by computerized dynamic posturography, and cognitive impairment and found a correlation between balance abilities and MMSE scores. They demonstrated a significant correlation between equilibrium scores and visuospatial and memory functions [22]. In an earlier study, Yogev and coworkers investigated only the relationship between gait ability and cognitive function. They found that executive function measures were significantly correlated with gait variability during dual tasking [23]. Allcock and colleagues, regarding cognitive abilities, investigated only attention skills and found an association between fall frequency and attention [3]. Williams and coworkers' study correlated motor abilities only with cognitive screening test scores, finding a relation between postural/gait instability and the MMSE [19].

Unlike these studies, more recent research has investigated other aspects of cognitive and motor performance in patients with PD and analyzed their correlations [20, 21]. Specifically, Domellöf and colleagues explored which aspects of cognition (memory, psychomotor speed, attention, language, visuospatial abilities, and executive functions) are connected to different motor signs as investigated by the UPDRS [20]. They found that bradykinesia was associated with executive functions (working memory and mental flexibility), whereas axial signs (such as postural instability, gait disturbances, and bulbar dysfunctions) were associated with memory and visuospatial abilities [20]. Similarly, Poletti and colleagues reported that the bradykinesia score on the UPDRS predicted performances on the executive tasks. But differently from Domellöf and colleagues' study, correlation analyses revealed that axial signs were also associated with executive deficits [21]. This result is in line with our data. Poletti and colleagues evaluated balance performance according to the UPDRS. It should be noted, however, that the UPDRS is a qualitative test that may be inadequate for accurately estimating balance and gait performance due to the subjective nature of the evaluation and the lack of normative criteria [38, 39]. Therefore, scales that yield a more varied estimate of posture and gait control are needed to obtain a better overall estimate of quantitative postural control, as we have done. Furthermore, this aspect could explain the differences in results between Domellöf and colleagues' and Poletti and coworkers' studies.

Similarly important is to extensively evaluate cognitive skills in patients with PD. In our view, the cognitive performance of patients with PD should be assessed by specific and appropriate cognitive measures in addition to the MMSE, considering that this examination alone does not allow identifying specific deficits in executive functions that are usually impaired in patients with PD [8–10].

Interestingly, our regression analyses showed a significant association between balance skills (as measured by the BBS) and executive functions (as measured by the FAB-it). From a rehabilitation point of view, it may be useful to clarify these aspects of the association between cognitive deficits and motor dysfunction in patients with PD in order to develop more appropriate rehabilitation programs that also include ecological situations for training motor and cognitive functions. In this context, prospective studies are needed to further investigate the effects of cognitive training on motor performance.

This study has several limitations. The main one is the small sample size that may have missed some aspects of the relationship between cognitive impairment and motor disorders in the correlation and regression analyses. Second, we excluded patients with MMSE score < 24; thus our population and results are limited to a relatively normal cognitive function for age. Third, we did not assess memory functions. Fourth, because we did not investigate patients in the “off” phase, we cannot draw conclusions about cognitive-motor relationships in the unmedicated state. Fifth, we did not include instrumental evaluations of gait and balance parameters.

## 5. Conclusions

Our findings have some clinical implications. Indeed, they may help early identification of cognitive deficits or motor dysfunctions in patients with PD who may benefit from rehabilitative strategies. Future prospective larger-scale studies including other instrumental motor outcomes are needed to strengthen our results and better explore the effects of training on cognitive-motor relationships in patients with PD.

## Figures and Tables

**Table 1:** Raw data of patients' performance in all outcomes.

FAB-it (0–18 points) Median (IQR)	14.00 (11.75; 16.25)
MoCA (0–30 points) Median (IQR)	22.00 (17.75; 25.25)
TMT-A (seconds) Mean (SD)	155.20 (122.55)
TMT-B (seconds) Mean (SD)	215.47 (103.07)
SVF (number of words) Median (IQR)	17.50 (15.25; 22.00)
MI (0–9 points) Median (IQR)	6.00 (3.00; 7.25)
BDI (0–63 points) Median (IQR)	13.00 (7.50; 18.00)
MMSE (0–30 points) Median (IQR)	29.00 (26.75; 30.00)
BBS (0–56 points) Median (IQR)	44.50 (38.50; 49.00)
10MWT (seconds) Mean (SD)	9.75 (4.65)
6MWT (meters) Mean (SD)	298.77 (96.80)
TUG (seconds) Mean (SD)	11.40 (4.84)
TUG-COG (seconds) Mean (SD)	11.96 (2.99)
UPDRS III (0–108 points) Median (IQR)	20.50 (16; 25.50)

SD, Standard Deviation; IQR, Interquartile Range; FAB-it, Frontal Assessment Battery-Italian version; MoCA, Montreal overall Cognitive Assessment; TMT, Trail Making Test; SVF: Semantic Verbal Fluency Test; MI, Memory with Interference; BDI, Beck Depression Inventory; MMSE, Mini Mental State Examination; BBS, Berg Balance Scale; 10MWT, 10-Meter Walking Test; 6MWT, 6-Minute Walking Test; TUG, Timed Up and Go; TUG-COG, Timed Up and Go under dual task condition; UPDRS III, Unified Parkinson's Disease Rating Scale, part III (motor examination).

**Table 2:** Correlation matrix for study variables (Spearman's rho).

Outcome measures	FAB-it	MoCA	TMT-A	TMT-B	SVF	MI	BDI	BBS	10MWT	6MWT	TUG	TUG-COG	UPDRS III
FAB-it	1.000												
MoCA	0.715^*^	1.000											
TMT-A	−0.517^*^	−0.776^*^	1.000										
TMT-B	−0.704^*^	0.822^*^	0.740^*^	1.000									
SVF	0.583^*^	0.778^*^	−0.579^*^	−0.729^*^	1.000								
MI	0.596^*^	0.664^*^	−0.511^*^	−0.553^*^	0.736^*^	1.000							
BDI	0.001	−0.106	0.223	0.030	0.133	−0.029	1.000						
BBS	0.790^*^	0.534^*^	−0.304	−0.597^*^	0.371	0.283	−0.114	1.000					
10MWT	−0.138	−0.090	0.114	0.308	−0.313	−0.146	−0.371	−0.169	1.000				
6MWT	0.149	0.058	−0.129	−0.095	0.292	0.082	−0.014	0.429	−0.715	1.000			
TUG	−0.372	−0.564^*^	0.477	0.630^*^	−0.713^*^	−0.243	−0.102	−0.499	0.762^*^	−0.709^*^	1.000		
TUG-COG	0.190	−0.667^*^	0.392	0.683^*^	−0.538	0.101	−0.192	−0.303	0.228	0.027	0.727^*^	1.000	
UPDRS III	−0.281	−0.035	−0.072	0.077	0.088	0.272	−0.103	−0.427	−0.029	0.135	0.074	0.606^*^	1.000

FAB-it, Frontal Assessment Battery-Italian version; MoCA, Montreal overall Cognitive Assessment; TMT, Trail Making Test; SVF, Semantic Verbal Fluency; MI, Memory with Interference Test; BDI, Beck Depression Inventory; BBS, Berg Balance Scale; 10MWT, 10-Meter Walking Test; 6MWT, 6-Minute Walking Test; TUG, Timed Up and Go Test; TUG-COG, Timed Up and Go Test under dual task condition; UPDRS III, Unified Parkinson's Disease Rating Scale part III (motor examination).

^*^Significant correlation (*P* < 0.05).

**Table 3:** Multiple linear regression analysis.

Dependent variables	Independent variables					
	FAB-it	MoCA	TMT-B	SVF	Age	BDI
BBS						
*β*	0.640	0.269	−0.117	−0.309	0.027	0.094
*P*	0.042^*^	0.481	0.745	0.322	0.892	0.635
TUG						
*β*	−0.661	−0.289	−0.086	0.126	−0.124	−0.084
*P*	0.259	0.704	0.908	0.814	0.748	0.821
TUG-COG						
*β*	0.148	−0.719	−0.229	−0.548	−0.322	0.005
*P*	0.690	0.223	0.679	0.320	0.307	0.991

FAB-it, Frontal Assessment Battery-Italian version; MoCA, Montreal overall Cognitive Assessment; TMT, Trail Making Test; SVF, Semantic Verbal Fluency; BDI, Beck Depression Inventory; BBS, Berg Balance Scale; TUG, Timed Up and Go Test; TUG-COG, Timed Up and Go Test under dual task condition.

^*^Significant correlation (P < 0.05).
